# Towards a better metabolic engineering reference: the microbial *chassis*

**DOI:** 10.1111/1751-7915.13363

**Published:** 2018-12-27

**Authors:** Kaleb Z. Abram, Zulema Udaondo

**Affiliations:** ^1^ Department of Biomedical Informatics University of Arkansas for Medical Sciences Little Rock AR 72205 USA

## Abstract

Highlight of the work of Calero and Nikel published in Microbial Biotechnology journal a few months ago.

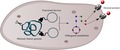

Almost two hundred years have passed since the birth of synthetic organic chemistry when the German chemist Friedrich Wöhler, performed the synthesis of urea in 1828 (Wöhler, 1828). Since then, our understanding of the properties and reactivity of organic molecules and the physical principles that compose living systems has progressed at a strong pace. This accumulation of knowledge over the years helped set the framework for the emergence of modern synthetic biology.

Synthetic biology consists of the manipulation and rationalization of complex biological processes to design or re‐purpose biological systems for useful applications (Purnick and Weiss, 2009). As with any engineering device, a certain level of abstraction is necessary to obtain a functional representation of a complex problem. For synthetic biology, the various biological components of the complex problem being investigated are represented in terms of *biological parts* or *devices*. These biological parts or devices could be ‘plugged‐in’ to a microbial chassis to address a given need. Once ‘plugged‐in’ to the chassis, these parts begin interacting under the corresponding genetic programme which encodes the target function.

With the assistance of genetic engineering, synthetic biology has rapidly advanced over the last few years for certain well‐characterized organisms such as *Escherichia coli* or *Bacillus subtilis*. However, one major challenge in this area is the dearth of diversity when choosing a platform organism to use as a *chassis*. Nevertheless, selection of a suitable host system is one of the main determinants of the technology, molecular tools, equipment and reagents needed to perform a chosen project. With the importance of suitable organism selection obvious, the selection should be made with the nature of the desired final product in mind at all times.

The work by Calero and Nikel (2019) reviews the predominant bacterial expression systems that have been successfully utilized in the field and the preferable properties that an ideal *chassis* should have for its use as a *cell‐factory* to avoid the non‐desirable drawbacks that could occur as a consequence of the expression of heterologous pathways in a given bacterial host. The ability to assemble protein‐coding sequences from different organisms into a single circuit would provide synthetic biologists with a considerable degree of flexibility in *chassis* selection for the design of novel systems. Unfortunately, it is difficult to realistically achieve this due to the notoriously complex nature of biological systems and the resultant difficulties in accurately predict the interactions between various components from different organisms, even in a system that is relatively well understood like for instance *E. coli*. For this reason, custom‐made reduced genomes harbouring only the genes required for its own replication and able to execute heterologous genes are such an attractive topic to biological engineers (Martínez‐Garcia and de Lorenzo, 2016).

The first major success in the quest for a functioning minimal genome was found in the whole‐genome design and synthesis of the synthetic genome JCVI‐syn1.0 (Hutchison *et al*., 2016). This synthetic genome was based on a *Mycoplasma mycoides* strain and contains 531 kbp and 473 genes. As Calero and Nikel highlight, the comparison of different versions of synthetic *Mycoplasma* revealed a minimal set of 265 genes needed for cell viability. Of these, almost one‐third of them encode for unknown functions which significantly reduces the direct application of a *chassis* based metabolic engineering process. These unknown functions in the minimal genome construct a knowledge gap that contains life essential biological pathways and functions.

Despite the various attempts at creating a minimal genome, our best efforts to replicate these genes of unknown function in other genomes can be described as ‘throwing genes at the wall and seeing what sticks’. Biological knowledge is still full of these small ‘black boxes’ where essential functions are carried out by unidentified pieces. Genetic engineers deal with these unknown elements under several approaches, such as modelling, which is key in the identification of elements that can reflect pivotal global properties with incomplete information.

In terms of metabolic network reconstruction, a ‘species level *chassis’* (Monk *et al*., 2013)*,* composed of metabolic genes that are shared by the entire species (or *core* metabolic genes; Udaondo *et al*., 2016, 2017), could be utilized to take advantage of the metabolic capabilities of strains that belong to species with ‘special abilities’ such as extremophiles. Thus, this approach could assist in the reconstruction of metabolic models for less characterized organisms by providing a collection of reference frameworks that can be used for draft reconstruction.

Despite the far reaching impacts of a *chassis* based approach, there stands a very clear limit to our progress that mirrors many other topics in the field of computational biology. As a field, we tend to focus on the well‐characterized species while ignoring the less characterized species due to the lack of available information. In turn, this investigation bias towards what we already have studied creates what could be described as a positive feedback loop. This loop is elliptical in shape due to the advances we make within a well‐characterized organism, but still is not the path effective and scalable research should be carried out on. It is critical for all fields in biology to break this cycle as quickly as possible. If we as a field can expand our knowledge to encompass a larger base area, the faster we can have meaningful and far impacting research. As a possible approach to bridge this gap, Calero and Nikel show the utility in the ‘bacterial chassis’ to rapidly expand our knowledge pool. They provide the reader with a quality review of the historical and current state of the art and provide useful insight into the future of the field.

## Conflict of interest

None declared.
